# Wear Mechanisms, Composition and Thickness of Antiwear Tribofilms Formed from Multi-Component Lubricants

**DOI:** 10.3390/ma17102324

**Published:** 2024-05-14

**Authors:** Anna E. Tsai, Kyriakos Komvopoulos

**Affiliations:** Department of Mechanical Engineering, University of California, Berkeley, CA 94720, USA

**Keywords:** antiwear tribofilm, base oil, dispersant, zinc dialkyl dithiophosphate, wear mechanisms

## Abstract

The antiwear properties of tribofilms formed on steel surfaces lubricated with various multi-component lubricants were investigated at an elevated temperature and under load-speed conditions conducive to sliding in the boundary lubrication regime. The lubricants contained base oil, reduced-level (secondary) zinc dialkyl dithiophosphate (ZDDP), and nitrogenous dispersant. The wear resistance of the tribofilms produced from different oil blends was evaluated in the context of the rate of change in the sliding track volume (wear rate for material loss) and the load-bearing capacity, chemical composition, and thickness of the tribofilms. Surface profilometry and scanning electron microscopy were used to quantify the wear performance and detect the prevailing wear mechanisms, whereas X-ray photoelectron spectroscopy elucidated the chemical composition and thickness of the tribofilms. The oil blends without ZDDP did not produce tribofilms with adequate antiwear properties, whereas the oil blends containing ZDDP and dispersant generated tribofilms with antiwear characteristics comparable to those of tribofilms produced from blends with a higher ZDDP content. Although dispersants can suspend oil contaminants and preserve the cleanness of the sliding surfaces, it was found that they can also reduce the antiwear efficacy of ZDDP. This was attributed to an additive-dispersant antagonistic behavior for surface adsorption sites affecting tribofilm chemistry and mechanical properties. Among the blends containing a mixture of ZDDP and dispersant, the best antiwear properties were demonstrated by the tribofilm produced from the blend consisting of base oil, 0.05 wt% ZDDP, and a bis-succinimide dispersant treated with ethylene carbonate. The findings of this investigation demonstrate the potential of multi-component lubricants with reduced-content ZDDP and nitrogen-based dispersant to form effective antiwear tribofilms.

## 1. Introduction

Interface interactions can significantly impact the functionality and endurance of a diverse range of contact-mode engineering components, such as bearings, gears, turbine blades, hard-disk drives, switches, and microelectromechanical systems, with each application requiring specific tailored surface treatments for inhibiting surface damage and material loss. A broadly used strategy for minimizing friction, wear, and heat generation at contact interfaces is to incorporate lubricants consisting of base oil and various additives, designed to impart specific surface functionalities by forming protective tribofilms and enhancing the stability and longevity of the lubricant. Tribofilms are usually sacrificial layers that are continuously produced by tribochemical reactions between the sliding surfaces and lubricant additives and depleted by different wear processes. Therefore, basic knowledge of tribofilm wear mechanisms is critical to mitigating wear at contact interfaces of boundary-lubricated systems.

The function of an additive is to form a low-friction, antiwear tribofilm through tribomechanically stimulated chemical action [1,2], such as shear stress-activated tribofilm formation [3,4]. Zinc dialkyl dithiophosphate (ZDDP) is one of the most common additives in lubricating oils. This is because of the exceptional effectiveness of ZDDP to form antiwear/antioxidant tribofilms on metal surfaces, which inhibit direct surface interaction at the contact interface. The mechanism of tribofilm formation in the presence of fully formulated oils containing ZDDP has been a topic of intensive research [5,6,7,8]. ZDDP affects boundary friction by undergoing absorption on rolling/sliding steel surfaces, with its outwardly oriented alkyl groups providing a shear plane similar to that produced by organic friction modifiers [9]. Tribofilm growth and adhesive bonding to the substrate surface can be influenced by the near-surface microstructure of the sliding surface [10], the substrate composition [11], the initial surface roughness [12], the applied contact load and lubricant volume [13], and other factors. ZDDP tribofilms usually comprise pad- or patchy-like flat regions separated by deep valleys [14,15,16], indicating tribofilm formation at the top of asperities where actual surface rubbing is encountered during sliding. There are several other antiwear/antioxidant additives besides ZDDP, such as molybdenum dithiocarbamate [17,18], cyclopropanecarboxylic acid and Ni nanoparticles [19], zinc oxide nanoparticles incorporated in diesel oil [20], 2D transition metal carbides, nitrides, and carbonitrides, known as MXenes [21], ionic liquids [22,23], over-based calcium sulfonate [24], multi-additive formulations consisting of oxygen-, nitrogen-, sulphur-, boron-, and molybdenum-containing components and metal deactivator [25,26], or combinations of ZDDP and molybdenum-complex additives [27].

Various microanalytical techniques have been used to characterize the tribofilm composition [28,29]. Tribofilms produced by various base oils containing ZDDP may demonstrate significant compositional differences. For example, polyalphaolefin base oil with ZDDP additive produces a tribofilm consisting of metal phosphates and sulfides and small amounts of carbon and iron oxides [30]. Phosphonium-phosphate ionic liquid with incorporated ZDDP leads to the formation of an amorphous tribofilm rich in iron, phosphorous, and oxygen with embedded debris consisting of an iron-rich core and an oxide shell, interfaced with the cast iron substrate through a hematite layer [22]. Other studies have shown the formation of amorphous ZDDP tribofilms consisting of zinc, oxygen, phosphorous, sulphur, and embedded magnetite nanoparticles [31], or small amounts of graphitic carbon, iron oxides, metal phosphates, and sulfide [30]. In view of the previous studies, a comprehensive analysis of the wear mechanisms of tribofilms must account for the tribofilm composition.

The identification of tribofilm wear mechanisms is further perplexed by antagonistic effects between additives and dispersants. Under certain conditions, the effectiveness of an additive could be reduced in the presence of a dispersant [31,32,33]. Dispersants are used to sustain the stability and cleanness of oil blends by inhibiting the agglomeration of wear debris [34], or nanoparticles used as lubricant additives [35]. For instance, different chemical interactions and tribological characteristics were found when detergents and dispersants were incorporated in a base oil that contained ZDDP [24,36,37,38]. Succinimides represent a common class of dispersants, whether simultaneously used with additives or alone in fully formulated lubricants [24,34,35,39]. These dispersants undergo adsorption on steel surfaces, forming nanometer-thick boundary films. Nevertheless, high dispersant concentration levels may suppress tribofilm formation [40], or the dispersant might become ineffective in reducing wear when the tribofilm is highly degraded because it could be entirely consumed in dispersing the wear debris [41]. However, while incorporating a succinimide dispersant in base oil can effectively disperse the wear debris, it cannot provide significant wear protection in the absence of an antiwear additive [34]. Chemical interactions between ZDDP and different nitrogenous dispersants have been reported to produce different friction behaviors, illuminating varying levels of competitive effects of these additives on friction characteristics [38]. Indisputably, understanding these antagonistic effects between additives and dispersants is critical for developing lubricant formulations that provide a balance between tribofilm antiwear properties and lubricant stability and cleanness.

The principal objective of this study was to investigate the antiwear characteristics of tribofilms formed on sliding steel surfaces lubricated with different types of oil blends, namely pure base oil, base oil containing either reduced-level ZDDP or a nitrogenous dispersant, and base oil with low-content ZDDP and nitrogenous dispersant. Sliding experiments were performed under boundary lubrication conditions to evaluate the wear characteristics of the tribofilms formed from different blends at elevated oil temperature. The efficacy of these blends to enhance the wear resistance through the formation of stable antiwear tribofilms is interpreted below in the context of surface profilometry and microanalytical results of the rate of change in sliding track volume (wear rate for material loss) and the load-bearing capacity, chemical composition, and thickness of the produced tribofilms.

## 2. Experimental Methods

### 2.1. Specimens and Wear Experiments

The ball-on-disk tribometer (Falex-6, thrust washer, Bruker Co., Billerica, MA, USA) used in a previous study of the friction characteristics of tribofilms formed from similar blends [38] was also used to perform the wear experiments of this study. Both the ball and disk specimens consisted of AISI 52100 steel (1.04 C, 0.35 Mn, 0.275 Si, 1.45 Cr, and 96.89 Fe, all wt%). The wear experiments were carried out under conditions of different loads in the range of 1.22–10.15 kg (i.e., 11.97 to 99.57 N), or mean Hertzian contact pressures in the range of 0.785–1.589 GPa (computed for 193 GPa elastic modulus, 0.3 Poisson’s ratio, and 8 cm ball diameter), oil temperature of 100 ± 5 °C, linear sliding velocity fixed at 0.19 m/s, and test time set at 2 h. Calculations confirmed that the foregoing test conditions resulted in sliding in the boundary lubrication regime. Further details about the properties, polishing, and cleaning of the specimens can be found elsewhere [38].

### 2.2. Blend Formulations

A total of seven blends were used in the experiments. Specifically, blend 1 consisted of base oil (100 N, group I), and played the role of the reference blend. The composition and analytical data of the base oil are given in Table 1, and its viscosity index is in the range of 80–120. Blends 2 and 3 consisted of base oil and reduced levels of pure, secondary ZDDP, i.e., 0.05 and 0.08 wt%, respectively, motivated by emerging developments for reducing phosphorous, sulfur, and sulfated ash. Blends 4 and 5 contained base oil and 0.1 wt% nitrogenous dispersant A (a bis-succinimide containing carbamate functionalities produced from a treatment with ethylene carbonate [42]) and 0.1 wt% nitrogenous dispersant B (produced by reacting a copolymer with at least one ether compound and at least one aromatic amine [43]), respectively. Dispersant A had a lower molecular weight and dispersancy than dispersant B. Blends 6 and 7 comprised base oil, 0.05 wt% ZDDP, and either 0.1 wt% dispersant A or 0.1 wt% dispersant B. In general, higher dispersant concentrations, i.e., in the range of 0.1–0.4 wt%, increase wear considerably [44]. Blends 4 and 6 contained significantly more nitrogen than blends 5 and 7. The chemical compositions of blends 1–7 are given in Table 2. The significant concentration of phosphorous, zinc, and sulphur in blends 6 and 7 is due to the ZDDP additive.

### 2.3. Surface Profilometry

The tested disk specimens were carefully cleaned with hexane to remove the oil residue without destroying the formed tribofilm on the sliding track. Then, the sliding tracks on the disks were examined with a mechanical stylus profilometer (Dektak IID, Veeco Instruments, NY, USA) with a spherical tip with a radius of curvature equal to 12.5 μm and vertical resolution equal to 0.1 nm. Cross-sectional profiles were obtained at various locations of each wear track by traversing the stylus tip perpendicular to the sliding direction at an average speed of 40 μm/s.

### 2.4. Wear Analysis

**Figure 1:**
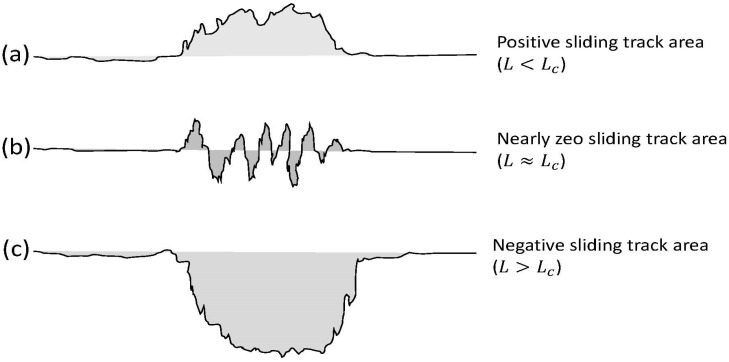
Schematics of typical profiles perpendicular to the sliding direction with (**a**) positive, (**b**) approximately zero, and (**c**) negative cross-sectional areas.

The surface profilometry measurements were used to quantify the wear behavior. A total of five cross-sectional profiles were obtained at five randomly selected locations of each sliding track, and the cross-sectional area of the particular sliding track was determined as the average of the measured cross-sectional areas. Figure 1 displays schematics of typical cross-sectional profiles of sliding tracks. A cross-sectional area was found to be positive, nearly zero, or negative, as shown in Figure 1a, Figure 1b, and Figure 1c, respectively, depending on the critical load *L_c_* of each tribofilm. A positive average cross-sectional area indicated the dominance of tribofilm formation (*L* < *L_c_*), a negative value implied the dominance of mechanical wear (*L* > *L_c_*), and a value close to zero suggested a balance between tribofilm formation and removal (*L* ≈ *L_c_*). Thus, the critical load corresponds to the transition from a nearly zero cross-sectional area to negative cross-sectional area (Figure 1). Henceforth, because a wear volume could not be obtained in all cases, the product of the average cross-sectional area and the circumference of the sliding track will be referred to as the change in sliding track volume *V*. The rate of change in sliding track volume (wear rate in those cases of material loss) $\dot{V}$ is defined as the ratio of *V* to the total sliding distance.

### 2.5. Microanalytical Techniques

The surfaces of the tested disk specimens were observed using a scanning electron microscope (SEM) (JSM-6700F, JEOL, Peabody, MA, USA). SEM micrographs provided insight into the formation and deterioration of the antiwear tribofilms and the dominant wear mechanism under various loads. After testing, the disk specimens were ultrasonically cleaned with heptane to remove the surface contaminants and oil residue. To enhance the SEM imaging quality, the accelerating voltage was set at 2 kV and imaging was performed in low secondary electron mode. To examine the wear characteristics, SEM micrographs were obtained at magnifications ranging from 150× to 10,000×. Relatively low magnifications provided an overall view of the sliding track features, whereas high magnifications revealed features intrinsic to tribofilm formation and the dominant wear mechanism.

Representative disk specimens tested under the lowest and highest load were examined using the SEM to determine the effect of the applied load on tribofilm formation. Disks tested at loads below and above the highest load that produced a stable nonzero contact voltage were also observed using the SEM. A total of 22 disk specimens representing 35 combinations of blend and load were used in the SEM studies.

The chemical composition and thickness of the tribofilms that formed on the sliding tracks were examined with an X-ray photoelectron spectroscopy (XPS) setup (Phi Quantera, Physical Electronics, Inc., Chanhassen, MN, USA) equipped with monochromatic Al-Kα X-rays (1486.6 eV). For comparison, XPS spectra were collected from surface regions on and off the sliding tracks, hereafter referred to as on- and off-scar spectra, respectively. To maximize the XPS signal, the diameter of the analyzed on-scar areas was varied between 100 and 200 μm, depending on the size of the feature examined. The same sampling area was used in the XPS analysis of adjacent off-scar surface areas. Elemental compositions were calculated as atomic percentages. Concentrations of less than 0.1 at% were considered to be at the noise level and were disregarded in the interpretation of the XPS results. Before the XPS analysis, each specimen was ultrasonically cleaned in light hydrocarbon solvent, such as heptane, to remove any oil residue or other surface contaminants. Chemical compositions were determined from the measured signal intensity normalized by empirical sensitivity factors. Chemical states were determined from line positions and reference data from the literature.

The tribofilm thickness was determined from elemental depth profiles obtained from the XPS analysis and in situ Ar^+^ ion milling using the same spectrometer. Specifically, a controlled amount of material was removed by Ar^+^ ion etching and the exposed surface was analyzed with the XPS. Iterative steps of etching and XPS analysis yielded the tribofilm elemental composition as a function of depth. The rate of depth profiling was calibrated with SiO_2_ films. For consistency, the tribofilm thickness was determined by the depth at which the total oxide content decreased to 50% of its maximum value. To avoid potential surface contamination effects, the elemental composition of each tribofilm was estimated at a depth equal to ~20% of the tribofilm thickness.

Similar to the disk specimens used in the SEM analysis, representative specimens were chosen for XPS analysis. Disk specimen selection was based on the lowest and highest load that, for a given blend, yielded a stable, nonzero contact voltage. Since the highest load was close to the critical load, more than one disks were selected for each blend due to the bi-modal contact voltage response (i.e., zero and nonzero contact voltage responses [38]) obtained when sliding occurred under the critical load. A total of 19 disks corresponding to 35 combinations of blend and load were used in the XPS studies.

## 3. Results and Discussion

### 3.1. Wear Rate

The wear process was classified as tribochemical in nature, resulting in the removal of both metal and tribofilm material from the sliding surfaces. The wear rate was influenced by the chemical reactivity of the interacting surfaces, which affected the rate of tribofilm formation (tribochemistry) and the intensity of surface tractions that controlled the loss of material (mechanical wear). Therefore, the sliding conditions (i.e., contact load, temperature, and sliding speed) had an immense consequence on the wear process. Tribochemical wear can be characterized by the critical load, *L_c_*, associated with the balance between the rate of tribochemical reactions resulting in tribofilm formation and the removal rate of the tribofilm by different wear mechanisms. Thus, the change in sliding track volume $\dot{V}$ was strongly affected by the applied load. In general, $\dot{V}$ > 0 for *L* < *L_c_*, $\dot{V}$ < 0 for *L* > *L_c_*, and $\dot{V}$ ≈ 0 (i.e., only surface roughening and negligible material loss) for *L* ≈ *L_c_*. Consequently, the wear behavior of different tribofilms can be discussed in terms of the variation in $\dot{V}$ with the applied load. Due to the large amount of data, the $\dot{V}$ versus *L* data were compared among blends assigned to the following three groups: group I: base oil with and without different concentrations of ZDDP (blends 1, 2, and 3); group II: base oil with dispersant A or B (blends 1, 4, and 5); group III: base oil containing 0.05 wt% ZDDP with or without dispersant A or B (blends 2, 6, and 7).

**Figure 2:**
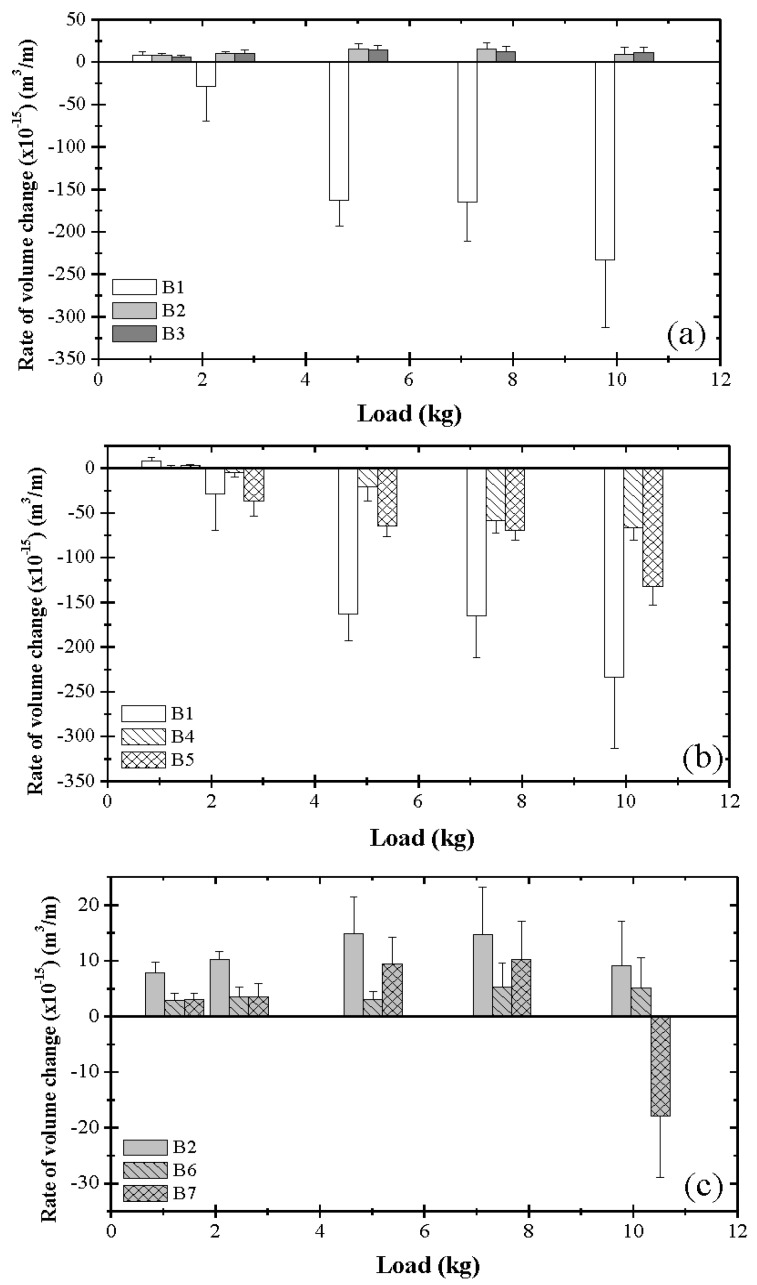
Rate of wear track volume change versus load for different blends: (**a**) B1 = blend 1 (base oil), B2 = blend 2 (base oil + 0.05 wt% ZDDP), and B3 = blend 3 (base oil + 0.08 wt% ZDDP); (**b**) B1 = blend 1 (base oil), B4 = blend 4 (base oil + 0.1 wt% dispersant A), B5 = blend 5 (base oil + 0.1 wt% dispersant B); (**c**) B2 = blend 2 (base oil + 0.05 wt% ZDDP), B6 = blend 6 (base oil + 0.05 wt% ZDDP + 0.1 wt% dispersant A); B7 = blend 7 (base oil + 0.05 wt% ZDDP + 0.1 wt% dispersant B).

Figure 2 shows wear rate data for different blends and various loads. While pure base oil (blend 1) did not protect the steel surfaces against wear ($\dot{V}$ < 0), tribofilm formation due to the ZDDP additive provided good wear protection ($\dot{V}$ > 0) over the entire load range (Figure 2a). The data for blends 2 and 3 illustrate the well-known antiwear character of tribofilms formed on steel surfaces sliding in the presence of ZDDP. The surfaces did not exhibit any material loss even for the highest load. However, the fairly similar $\dot{V}$ values of blends 2 and 3 for all loads indicate that the effect of ZDDP concentration on the tribofilm wear performance needs further investigation. The wear rate data of group II (Figure 2b) show that blends 4 and 5 produced tribofilms exhibiting better wear protection than that of the tribofilms due to blend 1 for *L* > 2.45 kg, whereas the tribofilm due to blend 1 yielded better wear protection for *L* = 1.22 kg. The highest wear rate due to the tribofilm produced by blend 1 is attributed to the abrasive action of oxide wear debris persisting at the contact interface for a longer time in the absence of a dispersant to quickly remove the debris from the ball/disk contact interface. Although blends 4 and 5 resulted in less wear than blend 1, they did not form protective antiwear tribofilms, as confirmed by contact voltage measurements [38]. It is also noted that the better dispersant did not provide a higher wear resistance. Although the better dispersancy of dispersant B is presumed to be the main reason for the lower friction coefficient obtained with blend 5 [38], the capacity of dispersant B to remove the wear debris might have increased the surface intimacy, resulting in more metal-to-metal contact during sliding, which, in turn, increased the wear rate. These results demonstrate that while a dispersant can reduce friction by dispersing and suspending particulates and other contaminants in the oil, it is not an effective wear inhibitor. Blends 6 and 7 from group III yielded $\dot{V}$ > 0 (Figure 2c), indicating the formation of antiwear tribofilms on the sliding tracks, consistent with contact voltage measurements [38], the only exception being blend 7 at the highest load. This can be attributed to the combined effects of high dispersancy and high surface tractions, which were not conducive to tribofilm replenishment, thus resulting in metal-to-metal contact and the dominance of mechanical wear. Even though the tribofilm due to blend 6 provided less wear protection than that due to blend 7, it protected the surfaces even under the highest load. Thus, blend 6 can be classified as a more effective wear inhibitor than blend 7. Despite the fact that the tribofilms due to blends 6 and 7 demonstrated good antiwear properties, the tribofilm due to blend 6 showed better wear protection than that due to blend 7, presumably due to the antagonistic roles of the ZDDP and dispersant B molecules, which diminished the capacity of blend 7 to form a tribofilm with the onset of sliding. Therefore, in view of the foregoing results, blend 6 may be inferred as the most promising antiwear blend.

Table 3 gives statistical data of $\dot{V}$ for different blends and loads. For the bimodal test conditions, instead of showing two $\dot{V}$ values, the average $\dot{V}$ of the two modes M1 and M2 was used for simplicity (mode M1 refers to the formation of a tribofilm exhibiting a stable steady-state contact voltage, whereas mode M2 refers to the formation of an unstable tribofilm producing a contact voltage fluctuating slightly above the zero level). For *L* = 2.45 kg, the calculated average $\dot{V}$ associated with blend 1 is equal to (−28.41 ± 41.46) × 10^−15^ m^3^/m; however, modes M1 and M2 gave $\dot{V}$ = (9.96 ± 2.8) × 10^−15^ m^3^/m and (–6.68 ± 2.05) × 10^−14^ m^3^/m, respectively. The negative and positive $\dot{V}$ values of modes M1 and M2 indicate that *L_c_* ≈ 2.45 kg for blend 1. The difference also explains the large standard deviation for this test condition. Blends 2, 3, 6, and 7 demonstrated the formation of antiwear tribofilms ($\dot{V}>$ 0), although blends 2 and 3 exhibited a higher load-bearing capacity. From the blends with reduced ZDDP concentration, the tribofilm due to blend 6 demonstrated the most promising antiwear capability.

### 3.2. Critical Load

Table 4 gives the critical load *L_c_* associated with each blend. For a given blend, *L_c_* was estimated by interpolating between the highest load that produced $\dot{V}$ > 0 and the lowest load that produced $\dot{V}$ < 0. Table 4 shows significantly lower *L_c_* values for the blends that did not contain ZDDP (i.e., blends 1, 4, and 5). These blends are classified as inadequate for wear protection. Conversely, blend 3 that contained 0.08 wt% ZDDP yielded *L_c_* > 10.15 kg. If the ultimate goal is to maintain $\dot{V}$ > 0 over the widest possible load range, then blends 2 and 6 are the most promising antiwear blends. If the results of the rate of change in sliding track volume (Table 3) and the critical load (Table 4) are considered together with the results of the steady-state coefficient of friction and the critical distance for stable tribofilm formation reported in a previous study [38], it could be argued that blend 2 might be slightly more advantageous from a wear protection perspective than blend 6; however, this advantage is not significant to draw a decisive conclusion.

### 3.3. Wear Mechanisms

The results presented hitherto demonstrate the efficacy of the examined oil blends to form antiwear tribofilms under certain contact conditions. Although the analysis of the wear behavior and the critical load for tribofilm failure provided important insight into competing tribochemical effects between the ZDDP additive and the dispersant as well as the load-carrying capacity of the formed antiwear tribofilms, understanding the dominant wear mechanisms requires further analysis. Hence, the dominant wear mechanisms of the tribofilms due to different blends are interpreted in this section in the context of microscopy results.

Adhesive and abrasive wear were common mechanisms contributing to the loss of material by tribochemical wear. Adhesive wear resulted in plucking off material from both sliding surfaces. The generated wear debris became trapped at the contact interface, causing the removal of material by a three-body abrasion process as debris rolled and abraded material from the sliding surfaces. The trapped wear debris could also anchor itself onto one of the surfaces, removing material from the opposed surface by a microcutting process similar to that caused by hard asperities in two-body abrasion. The longer the wear debris remained at the contact interface, the higher the wear rate. The role of an antiwear additive, such as ZDDP, is to protect the surfaces from the plowing or microcutting action of hard particles through the formation of a protective tribofilm, whereas the role of the dispersant is to prevent the wear debris from agglomerating and accessing the contact interface. In addition to microcutting and plowing, hard wear debris may also lead to material loss by the initiation and propagation of surface and/or subsurface cracks.

**Figure 3:**
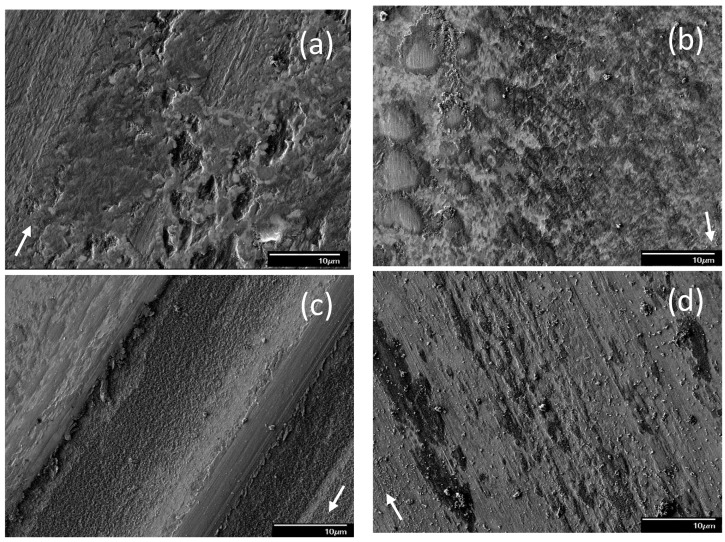
SEM micrographs of sliding tracks on steel surfaces lubricated with blend 1 (base oil) for a load equal to (**a**) 1.22, (**b**) 2.45 (mode M1), (**c**) 2.45 (mode M2), and (**d**) 10.15 kg (modes M1 and M2 indicate bimodal behavior; arrows indicate the sliding direction).

SEM micrographs of the sliding track topographies were used to identify the dominant wear mechanism(s) for different blend–load combinations. Figure 3 shows representative SEM images of sliding tracks on steel surfaces lubricated with pure base oil for different loads. For a light load (1.22 kg), the sliding track appears to be covered with layers of iron oxide (Figure 3a). The oxide tribofilm contains some pits, most likely caused by adhesive wear. Some portion of the tribofilm also possesses scratch marks oriented along the sliding direction, indicative of mild abrasive wear. Since the abrasive marks are not superficial, the protective tribofilm that formed under the certain test condition seems to be relatively soft. A bimodal behavior was observed for a load equal to 2.45 kg, with modes M1 and M2 yielding different wear features, as shown in Figure 3b and Figure 3c, respectively. The tribofilm for mode M1 demonstrated a “patchy” appearance, indicating that the removal of the iron oxide was predominantly due to adhesive wear. The tribofilm for this test condition was relatively hard, as evidenced by the formation of shallow wear marks, especially onto the large tribofilm patches. Alternatively, the tribofilm produced under mode M2 conditions exhibited many wear grooves of variable width along the sliding direction, with bare metal surface exposed in few of the grooves. The dominant wear mechanism for this test conditions was characterized by microplowing caused by three-body abrasive wear. Similar to mode M1, the light wear marks on the remaining tribofilm produced under mode M2 conditions indicated that this tribofilm was also relatively hard. The topographical differences between the two modes correlate with the difference in tribofilm formation distance and the $\dot{V}$ data obtained under these test conditions. The bare metal exposed in the wide grooves resulted in metal-to-metal contact, which explains the development of wear, i.e., $\dot{V}$ < 0 (Figure 2a) for mode M2 conditions under a 2.45 kg load. For a relatively high load (10.15 kg), there was minimal or no tribofilm remaining on the wear scar at the end of testing (Figure 3d). The tribofilm was stripped off from the wear track, suggesting a higher tribofilm removal rate than formation rate. The produced high contact pressure enhanced the establishment of metal-to-metal contacts at the sliding interface, resulting in the formation of more wear debris on the wear tracks.

**Figure 4:**
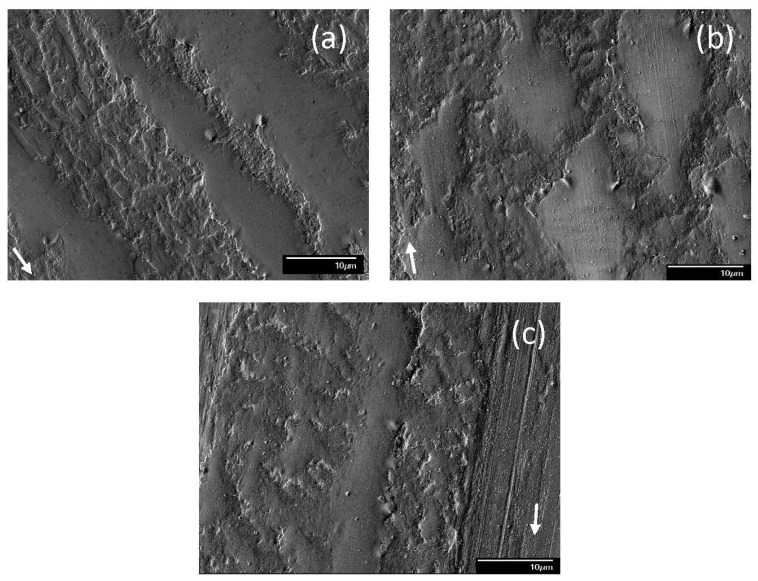
SEM micrographs of sliding tracks on steel surfaces lubricated with blend 2 (base oil + 0.05 wt% ZDDP) for a load equal to (**a**) 1.22, (**b**) 10.15 (mode M1), and (**c**) 10.15 kg (mode M2) (modes M1 and M2 indicate bimodal behavior; arrows indicate the sliding direction).

The incorporation of ZDDP in the base oil resulted in significantly different surface morphologies. Figure 4 shows representative surface features attributed to the tribofilm due to blend 2. For a light load (1.22 kg), the sliding track demonstrated patch-filled grooves between the strips of a lightly scratched tribofilm (Figure 4a), indicating the simultaneous operation of two or more wear mechanisms and the formation of a rather hard tribofilm. The presence of grooves between the partially stripped tribofilm can be attributed to microcutting by wear debris trapped at the contact interface. The patch-like tribofilm inside the grooves may be associated with the combined effects of adhesive wear and the localized replenishment of the tribofilm. Moreover, microcracking was observed along the edges of the tribofilm strips. A bimodal behavior occurred for sliding under a high load (10.15 kg) in the presence of blend 2. Mode M1 showed mostly patch-like tribofilm formation, attributed to the synergism of adhesive wear, localized tribofilm replenishment, and microcracking along the patch edges (Figure 4b). Conversely, mode M2 showed distinct groove formation along the sliding direction (Figure 4c). Contrary to light-load surface features, the mode M2 behavior displayed in the presence of blend 2 was characterized by pitted tribofilm strips with grooves filled with a soft tribofilm, as evidenced by the formation of deep and continuous wear scars. These continuous grooves and wear scars are distinctive surface features created by the plowing action of wear debris and strain hardened asperities, whereas the pitted tribofilm strips are indicative of adhesive wear. The difference in surface morphologies of the two modes may be associated with differences in tribofilm thickness, which might be correlated with the distance needed to form a coherent tribofilm. A longer sliding distance (time) for the formation of an antiwear tribofilm may also be associated with the development of a thinner tribofilm that was less sustainable and, therefore, more susceptible to wear.

**Figure 5:**
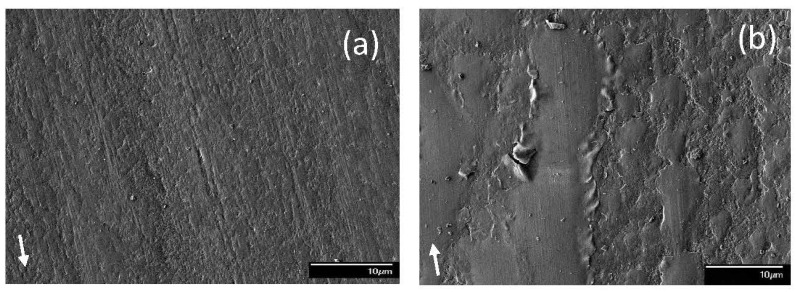
SEM micrographs of sliding tracks on steel surfaces lubricated with blend 3 (base oil + 0.08 wt% ZDDP) for a load equal to (**a**) 1.22 and (**b**) 10.15 kg (arrows indicate the sliding direction).

Under the effect of a light load (1.22 kg), blend 3 produced a sustainable tribofilm on the wear track (Figure 5a). Even though the tribofilm exhibited a few micropits and minor wear scars, it was well-preserved in general. Compared to the tribofilm formed from blend 2, it appears that the higher concentration of ZDDP in blend 3 was beneficial to the faster replenishment of the tribofilm, consequently yielding better wear protection. However, in the case of a high load (10.15 kg), the tribofilm delaminated and was removed from the surface by adhesive wear, resulting in a patch-like surface morphology (Figure 5b). In addition, microcracking due to the high local stress was also encountered along the tribofilm edges. Similar to the tribofilm produced by blend 2 during sliding under a high load (Figure 4b), the tribofilm produced by blend 3 appeared to be hard, as indicated by the light scratch marks. A comparison of Figure 4 and Figure 5 indicates that the tribofilm due to blend 3 demonstrated better protection against sliding wear than the tribofilm due to blend 2.

**Figure 6:**
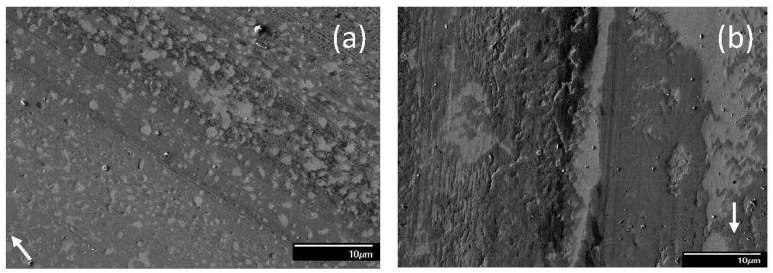
SEM micrographs of sliding tracks on steel surfaces lubricated with blend 4 (base oil + 0.1 wt% dispersant A) for a load equal to (**a**) 1.22 and (**b**) 10.15 kg (arrows indicate the sliding direction).

**Figure 7:**
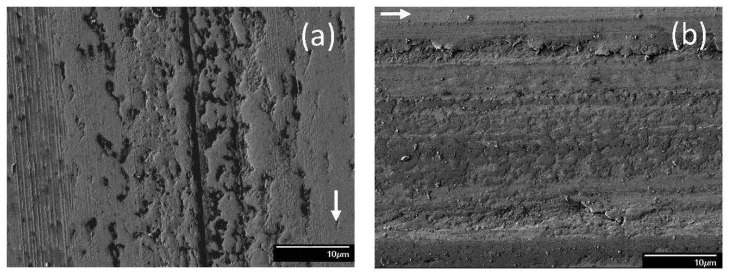
SEM micrographs of sliding tracks on steel surfaces lubricated with blend 5 (base oil + 0.1 wt% dispersant B) for a load equal to (**a**) 1.22 and (**b**) 10.15 kg (arrows indicate the sliding direction).

Figure 6 and Figure 7 show representative surface morphologies for blends 4 and 5, respectively. For a light load (1.22 kg), the dominant wear mechanism displayed the characteristics of adhesive wear for both blends (Figure 6a and Figure 7a). Blend 4 resulted in shallow pockets dense in population, whereas blend 5 produced deep cavities spread out on the wear track. In addition, surface scratches were relatively shallower for blend 4 than blend 5. For sliding under a high load (10.15 kg), the steel surfaces lubricated with blend 4 demonstrated the formation of shallow plowing grooves (Figure 6b), while those lubricated with blend 5 showed evidence of microcutting (Figure 7b), suggesting that microscale abrasion played a dominant role in the wear process. The different wear mechanisms are consistent with the slightly better wear protection associated with blend 4 than blend 5 at all loads (Figure 2b). This is because microscale plowing does not actually contribute to material loss as the material is not removed, rather it is displaced to the sides of the plowing grooves. Alternatively, microscale cutting removes material in the form of wear debris, consequently resulting in material loss.

**Figure 8:**
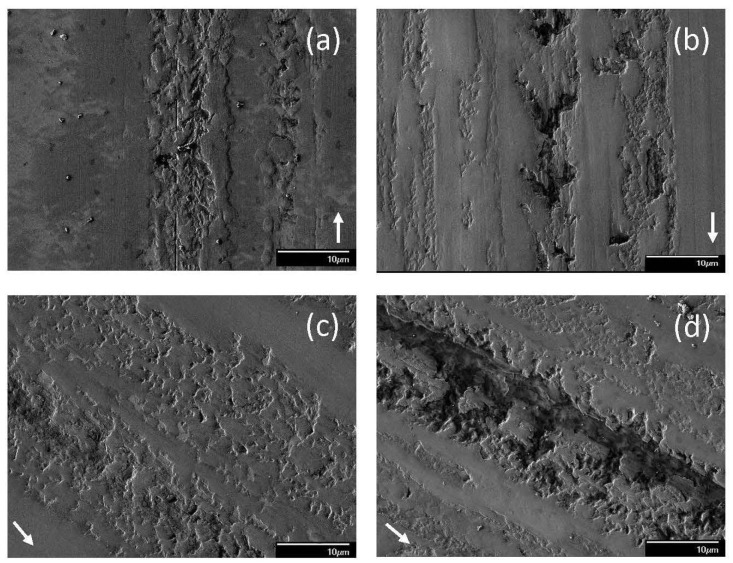
SEM micrographs of sliding tracks on steel surfaces lubricated with blend 6 (base oil + 0.05 wt% ZDDP + 0.1 wt% dispersant A) for a load equal to (**a**) 1.22, (**b**) 7.49 (mode M1), (**c**) 7.49 (mode M2), and (**d**) 10.15 kg (modes M1 and M2 indicate bimodal behavior; arrows indicate the sliding direction).

When dispersants were mixed with blends containing 0.05 wt% ZDDP, the resulting mixtures increased the complexity of the tribochemical reactions leading to the formation of antiwear tribofilms. Typical wear features on the steel surfaces lubricated with blend 6 showed that adhesive wear was the dominant mechanism under various loads (Figure 8). Surface micropits with different sizes, shapes, and population densities appeared on the wear tracks. Mild surface damage was observed for a light load (1.22 kg) encompassing material smearing and microscopic dimples (Figure 8a). A bimodal behavior was encountered when an intermediate load (7.49 kg) was applied. The main difference between modes M1 and M2 was the characteristics of pit formation. Mode M1 produced fewer but larger in size and deeper in depth pits (Figure 8b) than mode M2 (Figure 8c). It also appeared that mode M1 generated a softer tribofilm than mode M2 because the scratch marks were more noticeable on the wear tracks produced under mode M1 conditions. For a high load (10.15 kg), deep plowing grooves dominated the wear track topography (Figure 8d). In all loading cases, the smooth plateaus illustrated the presence of an unworn tribofilm that survived rupture by the sliding process.

**Figure 9:**
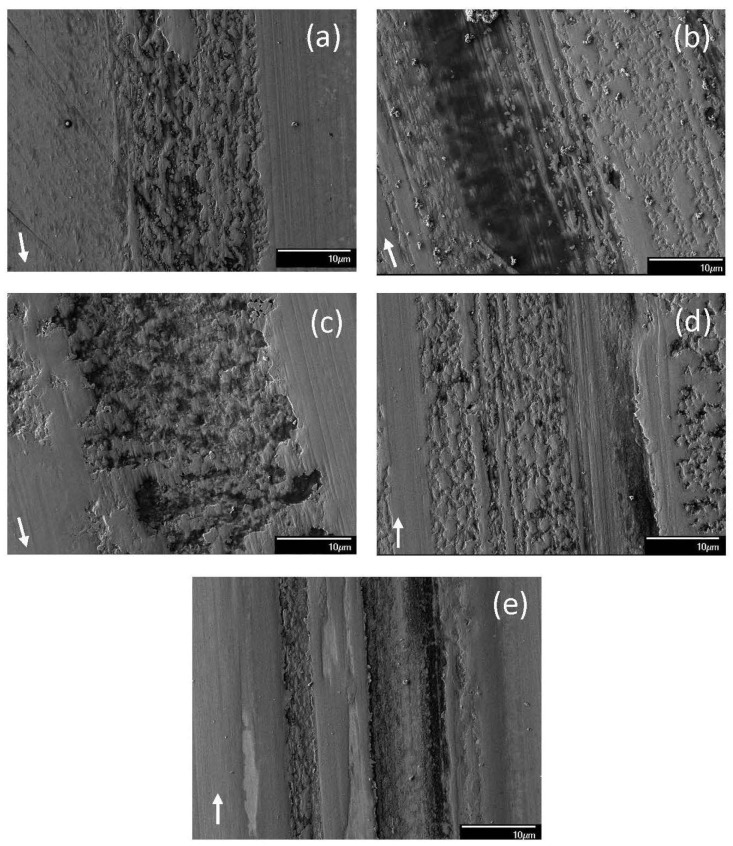
SEM micrographs of sliding tracks on steel surfaces lubricated with blend 7 (base oil + 0.05 wt% ZDDP + 0.1 wt% dispersant B) for a load equal to (**a**) 1.22, (**b**) 7.49 (mode M1), (**c**) 7.49 (mode M2), (**d**) 10.15 (mode M1); (**e**) 10.15 kg (mode M2) (modes M1 and M2 indicate bimodal behavior; arrows indicate the sliding direction).

The dominant wear mechanism of the tribofilm formed from blend 7 demonstrated a load dependence (Figure 9). For a 1.22 kg load, adhesive wear was the dominant mechanism as evidenced by the presence of numerous microdimples on the tribofilm surface (Figure 9a). A bimodal behavior was observed for both 7.49 and 10.15 kg loads. For a 7.49 kg load, the surface features suggested the formation of a softer tribofilm under mode M1 (Figure 9b) than mode M2 (Figure 9c) conditions, as revealed by the deeper wear track for mode M2. These surface features suggest that the principal wear mechanism under mode M1 sliding was microplowing, resulting in the formation of deep grooves and the removal of the patchy top layer of the tribofilm. Mode M2 displayed predominantly adhesive wear, with the removal of large patches of the tribofilm creating numerous pits accompanied by localized tribofilm formation. For a 10.15 kg load, mode M1 wear was mainly due to microplowing and adhesive wear (Figure 9d); however, the same test conditions also resulted in wear dominated by microcutting, which was characterized as mode M2 (Figure 9e). If a groove was too wide, the time for the chemical reaction to reform the protective tribofilm might have been insufficient, leaving bare metal exposed at the ball/disk contact interface.

The SEM micrographs shown above illustrate the contribution of different blends and loading conditions to the sliding track topographies. The protective tribofilms were usually heterogeneous with distinct localized features, especially at the microscale, indicating that tribofilm formation and tribofilm removal were localized processes.

### 3.4. Tribofilm Chemical Composition

The chemical composition of the tribofilms was examined to gain a better understanding of their origin. Specifically, XPS analysis of the sliding tracks produced under various loads was performed to shed light onto tribofilm formation from different blends. A comprehensive description of the surface elemental compositions at on- and off-scar locations obtained by XPS for different blends and loads can be found in Section SI of Supplementary Material (SM). Moreover, detailed information about the chemical states of carbon, sulfur, and iron at both on- and off-scar locations are given in Section SII of SM for various blends and loads. The chemical composition is presented as an elemental concentration in units of atomic percentage. An elemental concentration was considered only if it was above 0.1 at%; otherwise, it was discarded as noise. It is noted that, although elemental concentrations provided a means for comparing the relative composition among sampling areas, a concentration cannot be used as an absolute quantity of the particular element in the tribofilm.

The presence of chlorine and calcium is attributed to surface contaminants because these elements were not part of the test materials and were relatively low in concentration among all the samples. The on-scar concentration of silicon was about 10% or less for blend 1 and less than 1.8% for all other blends. While silicon was part of the chemical composition of the steel specimens, it only contributed to tribofilm formation during sliding in the absence of the additive. Although carbon made up a good portion of the tribofilm’s top surface, it was considered to be adventitious because most of it was organic. Oxygen was another significant contributor to the tribofilm composition because the tribofilms mainly consisted of various oxides. Even though chlorine, calcium, silicon, carbon, and oxygen existed in the tribofilms, tribofilm characterization was better performed by using additive elements, such as nitrogen, phosphorus, sulfur, iron, and zinc. Therefore, the foregoing elements were used to analyze the chemical composition of the tribofilms for various blends and loads (Table 5).

Nitrogen was usually found in forms of amine. Special interest was placed on nitrogen because both dispersants contained nitrogen in their functional groups. Phosphorus was usually present as phosphate, a glassy compound that provided the principal antiwear protection. Sulfur typically possessed two chemical states, i.e., sulfide and sulfate. The atomic percentage of each chemical state of sulfur was determined by curve fitting. Sulfide, which was more reduced than sulfate at on-scar locations, is known to generate a softer tribofilm that reduces friction. Iron existed at the top surface of the tribofilms in the form of iron oxides, formed without the added energy of the sliding process. Zinc was a good indicator of the existence of phosphate in the tribofilms because it is known to act as one of the cations that stabilizes phosphate and forms zinc phosphate glass under boundary lubrication sliding conditions and in the presence of ZDDP.

The concentration of amine (nitrogen) was relatively consistent among the test conditions; however, nitrogen increased under high loads in the presence of blend 6. Since dispersant A was saturated with nitrogen, nitrogen-containing compounds were liberated from the dispersant under the effect of the high contact pressure generated at high loads, making them available contributors for tribofilm formation. Phosphate is known to be a glassy hard phase that enhances antiwear behavior. The on-scar concentration of phosphorus was high in the tribofilms formed from blends containing ZDDP, in agreement with the shorter distance for tribofilm formation or $\dot{V}$ > 0 observed with blends 2, 3, 6, and 7. Moreover, a ZDDP-derived tribofilm was usually characterized by a high phosphorus concentration at high loads, implying that ZDDP-derived tribofilms required additional energy from the sliding process. This observation is also confirmed by the variation in $\dot{V}$ with load for the blends containing ZDDP (blends 2, 3, 6, and 7).

On-scar locations exhibited higher sulfur concentrations than off-scar locations. The difference in concentration suggested mechanical energy input was essential for the formation of sulfur-derived tribofilms. Sulfide is known to reduce friction. In general, the blends that contained ZDDP demonstrated a high on-scar sulfide concentration, consistent with the lower steady-state friction coefficients that characterized these blends [38]. The iron detected on the surfaces was usually in the form of iron oxides, which have low activation energy because their formation does not require additional work, such as the external work supplied by the normal and shear surface tractions. As expected, off-scar locations showed a higher concentration of iron than on-scar locations. In general, the iron concentration was lower in the tribofilms that were rich in phosphorus, sulfur, and zinc. For example, the iron concentration in the tribofilms formed from blends 1, 4, and 5 that did not contain ZDDP was generally higher than that in the tribofilms formed from blends 2, 3, 6, and 7. Even in blends with ZDDP, the iron concentration was high for a light load, implying insufficient activation energy to form ZDDP-derived tribofilms under light-load sliding conditions. It appears that an effective ZDDP-derived tribofilm must contain zinc, phosphorus, and sulfur; however, the relative amounts of each element in the tribofilm differed from that in the original molecular ratio of 1:2:4. The concentration of zinc followed a pattern fairly analogous to that of phosphate. An exception was noted for blend 6 under a high load. The decrease in zinc and the increase in nitrogen concentration occurred simultaneously under the same test conditions, suggesting that competition between ZDDP and dispersant compromised the tribofilm chemical composition.

### 3.5. Tribofilm Thickness

Composition depth profiles for various blends and loads were obtained from XPS measurements. Depth profiles of amine, phosphate, sulfide, iron, iron oxide, zinc, oxygen, carbide, and potassium for each blend can be found in Section SIII of SM. The tribofilm thickness was determined by the depth at which the oxide concentration decreased to 50% of its maximum value (Table 6). In the absence of ZDDP (blends 1, 4, and 5), iron-oxide tribofilms of average thickness equal to ~80 and ~90 nm formed under the lightest load (1.22 kg). Depending on the mode of tribofilm formation in the presence of blend 1, increasing the load to 2.45 kg yielded either an increase (mode M1) or a decrease (mode M2) in tribofilm thickness, i.e., ~240 and ~40 nm, respectively. A sustainable tribofilm formed rapidly under mode M1, whereas tribofilm formation was not observed under mode M2. The difference in the critical distance for tribofilm formation was confirmed by the results of the rate of change in sliding track volume (wear rate for material loss) and tribofilm thickness. Under the lightest load, blend 2 produced a tribofilm of thickness equal to ~160 nm. Similar to blend 1, the mode of tribofilm formation was affected by increasing the load to 10.15 kg, i.e., a critical distance for tribofilm formation equal to ~230 nm (mode M1) and ~170 nm (mode M2). Variation in the mode of tribofilm formation also affected the critical sliding distance for the development of a stable tribofilm and, consequently, the wear rate. Comparing the two modes, mode M1 resulted in thicker and harder tribofilms that provided better wear protection. The tribofilm chemical compositions suggest that different types of tribofilm formed under low and high loads in the presence of blend 3. In general, the load increase intensified the surface tractions, resulting in a greater energy supply for activating the formation of a more effective antiwear tribofilm. Incorporating a dispersant in the same blend with ZDDP further complicated tribofilm formation. However, based on the tribofilm thickness results, the tribofilm produced by blend 6 provided better wear protection than that produced by blend 7 because of the monotonic increase of the tribofilm thickness with the load. The decrease in tribofilm thickness for blend 7 under a high load indicated a reduced wear resistance, consistent with the fact that $\dot{V}$ < 0 for blend 7 at a 10.15 kg load (Figure 2c). Therefore, it may be inferred that, from the tribofilm thickness standpoint, blends 2 and 6 demonstrated the highest potential to inhibit wear.

The novelty of the present work is the insight provided into the wear characteristics, load-carrying capacity, composition, and thickness of tribofilms produced from various multi-component lubricants with reduced contents of secondary ZDDP and a very small fraction of different nitrogenous dispersants. The presented results and discussion indicate that a plausible strategy for reducing the portion of additives that produce hazardous emissions, such as ZDDP, is to replace a significant portion of these additives by other substances that are not harmful to the environment and do not poison emission-control systems while maintaining adequate wear protection.

## 4. Conclusions

The formation of antiwear tribofilms from different blends containing base oil, reduced levels of secondary ZDDP, and different types of nitrogenous dispersants was evaluated in the context of wear experiments performed with steel samples at an elevated oil temperature and under load-speed conditions conducive to sliding in the boundary lubrication regime. The results of the rate of change in sliding track volume (wear rate for material loss) obtained from surface profilometry measurements, SEM imaging revealing the dominant tribofilm wear mechanisms, and XPS analysis of the chemical composition and thickness of tribofilms produced from different blends were used to assess the wear characteristics of the tribofilms during sliding under various loads. Based on the presented results and discussion, the following main conclusions can be drawn from this study.

(1) In the case of lubrication by pure gear base oil, the surface oxide film deteriorated under high-load sliding conditions.
(2) ZDDP suppressed wear even for sliding under the highest load used in this study. Nevertheless, the activation energy required for the formation of an iron oxide tribofilm was much lower than that of a ZDDP-derived tribofilm.
(3) Although the coexistence of dispersant and ZDDP in base oil, i.e., blend 6 (base oil + 0.05 wt% ZDDP + dispersant B), resulted in the formation of a tribofilm exhibiting lower wear resistance than the tribofilms produced from blends consisting of base oil and ZDDP, material loss did not occur throughout the entire load range.
(4) The tribofilm produced from blend 7 (base oil + 0.05 wt% ZDDP + dispersant B) demonstrated better wear behavior under low- and intermediate-load sliding conditions; however, the critical load of this tribofilm was lower than that of the tribofilm produced from blend 6.
(5) Among all the blends, the tribofilms due to blends 2 and 6 demonstrated overall better wear characteristics.
(6) Tribochemical wear of the tribofilms was characterized by the dominance of one or more wear mechanisms. The most frequently encountered wear mechanism was adhesive wear, resulting in tribofilm cohesive failure. Different types of abrasive wear were demonstrated by some of the tribofilms. Tribofilm replenishment was generally characterized by the formation of patch-like patterns on the sliding track.
(7) Tribofilms of iron oxide possessed an average thickness of ~80 nm, whereas ZDDP-derived tribofilms were characterized by a larger thickness of ~200 nm.
(8) The findings of this study provide impetus for further investigations aimed at replacing environmentally harmful additives with other substances that are not harmful to the environment and can form antiwear tribofilms.

## Figures and Tables

**Table 1:** Composition and analytical data of base oil (Group I) *.

Composition	Content (wt%)
Aromatics	24.8
Polars	1.4
Saturates	73.8
Insolubles	0.0
Analytical Data	
Element	Amount (ppm)
B	<1.8
Ca	<0.9
K	<2.6
P	<6.1
S	4040
Zn	<0.9
N	76.88

* Quoted by Chevron Oronite Co., Richmond, CA, USA.

**Table 2:** Chemical composition of blends.

Blend	Composition	Concentration (ppm)			
		N	P	Zn	S
1	base oil	–	–	–	19
2	base oil + 0.05 wt% ZDDP	1.7	513	545	1042
3	base oil + 0.08 wt% ZDDP	–	803	868	1615
4	base oil + 0.1 wt% dispersant A	894	–	–	57
5	base oil + 0.1 wt% dispersant B	175	–	–	26.7
6	base oil + 0.05 wt% ZDDP + 0.1 wt% dispersant A	888	516	552	1102
7	base oil + 0.05 wt% ZDDP + 0.1 wt% dispersant B	175	519	551	1081

**Table 3:** Wear rate (×10^−15^) (m^3^/m) for different blends and loads.

Blend	Load (kg)				
	1.22	2.45	5.02	7.49	10.15
1	7.86 ± 4.43	−28.41 ± 41.46	−163.05 ± 29.63	−165.23 ± 46.17	−233.53 ± 79.53
2	7.86 ± 1.87	10.28 ± 1.44	14.80 ± 6.63	14.77 ± 8.45	9.12 ± 8.01
3	5.72 ± 2.39	9.86 ± 4.20	14.04 ± 6.09	12.66 ± 6.31	11.72 ± 5.47
4	1.25 ± 1.57	−4.63 ± 5.11	−21.08 ± 15.59	−58.61 ± 14.18	−66.35 ± 13.82
5	2.71 ± 1.19	−36.58 ± 16.65	−64.76 ± 11.93	−69.75 ± 10.33	−132.32 ± 20.50
6	2.93 ± 1.23	3.58 ± 1.79	3.08 ± 1.38	5.35 ± 4.23	5.17 ± 5.33
7	3.02 ± 1.18	3.58 ± 2.32	9.38 ± 4.83	10.20 ± 6.87	−17.88 ± 10.97

**Table 4:** Critical load for different blends.

Blend	Composition	Critical Load (kg)
1	base oil	~2.45
2	base oil + 0.05 wt% ZDDP	>10.15
3	base oil + 0.08 wt% ZDDP	>10.15
4	base oil + 0.1 wt% dispersant A	~1.22
5	base oil + 0.1 wt% dispersant B	~1.22
6	base oil + 0.05 wt% ZDDP + 0.1 wt% dispersant A	>10.15
7	base oil + 0.05 wt% ZDDP + 0.1 wt% dispersant B	~9.0

**Table 5:** Tribofilm chemical composition for different blends and loads.

Blend	Load (kg) (Mode)	Surface Location	Element Concentration (at%)						
			Amine (N)	P	S	Sulfide	Sulfate	Fe	Zn
1	1.22	on-scar	2.36	0.76	0.27	−	0.27	2.40	−
		off-scar	2.94	−	0.62	0.15	0.47	4.74	−
	2.45 (M1)	on-scar	1.16	1.86	0.33	0.10	0.23	3.31	0.55
		off-scar	1.41	−	0.17	0.01	0.16	6.48	0.21
	2.45 (M2)	on-scar	0.47	0.63	0.10	0.01	0.09	7.60	0.15
		off-scar	0.58	0.15	0.10	−	0.10	9.27	0.13
2	1.22	on-scar	2.56	5.38	3.06	2.56	0.50	3.45	6.26
		off-scar	1.00	0.87	0.93	0.36	0.57	6.96	4.31
	10.15 (M1)	on-scar	2.98	11.24	2.06	2.06	−	−	11.60
		off-scar	1.94	1.29	1.30	0.65	0.65	6.77	4.07
	10.15 (M2)	on-scar	2.85	10.07	2.56	2.51	0.05	0.82	9.66
		off-scar	1.39	0.77	1.07	0.48	0.59	6.66	2.50
3	1.22	on-scar	2.04	4.85	3.71	3.39	0.32	2.28	5.76
		off-scar	1.67	0.40	0.93	0.46	0.47	4.37	1.91
	10.15	on-scar	2.54	10.11	2.36	2.36	0.00	0.17	11.91
		off-scar	3.91	0.40	1.50	1.00	0.50	4.51	4.95
4	1.22	on-scar	2.84	0.94	0.46	0.07	0.39	6.96	0.16
		off-scar	3.97	−	0.39	0.17	0.22	5.95	−
5	1.22	on-scar	1.38	0.99	−	−	−	3.56	−
		off-scar	3.07	0.18	−	−	−	5.90	−
6	1.22	on-scar	2.68	2.30	2.40	2.27	0.13	3.69	5.51
		off-scar	3.28	0.11	0.25	−	0.25	7.15	0.79
	7.49 (M1)	on-scar	4.87	11.40	6.39	6.19	0.20	0.58	0.47
		off-scar	5.17	0.41	0.56	0.12	0.44	2.62	0.14
	7.49 (M2)	on-scar	5.16	7.49	4.03	4.03	−	0.45	0.86
		off-scar	4.60	0.45	0.12	0.11	0.01	4.61	0.81
	10.15	on-scar	4.44	8.41	3.96	3.84	0.12	0.92	2.40
		off-scar	5.37	0.31	1.01	0.64	0.37	4.06	0.64
7	1.22	on-scar	3.47	4.20	2.47	2.30	0.17	1.59	2.69
		off-scar	2.24	0.43	0.32	0.12	0.20	4.77	0.91
	7.49 (M1)	on-scar	2.37	7.39	2.88	2.85	0.03	1.22	6.64
		off-scar	2.46	0.40	0.39	0.13	0.26	3.56	0.81
	7.49 (M2)	on-scar	1.50	8.34	2.98	2.77	0.21	1.36	12.53
		off-scar	1.38	0.16	0.15	−	0.15	8.83	1.79
	10.15 (M1)	on-scar	1.68	7.46	2.62	2.38	0.24	1.39	8.51
		off-scar	2.84	0.35	0.64	0.10	0.54	5.29	1.16
	10.15 (M2)	on-scar	1.58	7.94	2.62	2.48	0.14	2.15	8.48
		off-scar	2.69	0.44	0.95	0.31	0.64	3.99	1.56

**Table 6:** Tribofilm thickness versus blend and load.

Blend	Load (kg)	Tribofilm Thickness (nm)
1	1.22	80
	2.45 (M1)	240
	2.45 (M2)	40
2	1.22	160
	10.15 (M1)	230
	10.15 (M2)	170
3	1.22	110
	10.15	230
4	1.22	80
5	1.22	90
6	1.22	30
	7.49 (M1)	200
	7.49 (M2)	220
	10.15	230
7	1.22	80
	7.49 (M1)	240
	7.49 (M2)	230
	10.15 (M1)	140
	10.15 (M2)	180

## Data Availability

Data are contained within the article and in the Supplementary Materials.

## Supplementary Materials

The following supporting information can be downloaded at https://www.mdpi.com/article/10.3390/ma17102324/s1: Section SI. Elemental composition (surface elemental compositions for on- and off-scar locations for various blends and loads); Section SII. Chemical composition (on- and off-scar surface chemical compositions for various blends and loads); Section SIII. Elemental depth profiles (elemental depth profiles for various blends and loads).
