# Supplementary material for: Wear Mechanisms, Composition and Thickness of Antiwear Tribofilms Formed from Multi-Component Lubricants
Source: Materials (Basel). 2024 May 14;17(10):2324. doi: 10.3390/ma17102324 (PMC11122858; doi:10.3390/ma17102324)
Supplement: Supplementary file 1 [file materials-17-02324-s001.zip › materials-2995318-supplementary.pdf]

Supplementary Material

SI. Elemental composition

Surface elemental compositions for on- and off-scar locations for various blends and loads are listed in the following table.

Contact Conditions		Surface	Element Concentration (at%)									
Blend	Load (kg)	Location	C	N	O	Si	P	S	Cl	Ca	Fe	Zn
Blend 1	1.22	On-Scar	46.7	2.4	33.9	10.6	0.8	0.3	0.1	0.4	2.4	--
		Off-Scar	52.2	2.9	32.8	1.3	--	0.6	0.1	0.3	4.7	--
Blend 1	2.45 (Mode 1)	On-Scar	36.94	1.16	43.45	8.96	1.86	0.33	--	0.11	3.31	0.55
		Off-Scar	45.52	1.41	38.43	0.57	--	0.17	--	0.62	6.48	0.21
Blend 1	2.45 (Mode 2)	On-Scar	33.52	0.47	45.49	4.19	0.63	0.1	--	0.21	7.6	0.15
		Off-Scar	36.75	0.58	42.57	0.69	0.15	0.1	--	0.49	9.27	0.13
Blend 2	1.22	On-Scar	34.87	2.56	40.21	0.39	5.38	3.06	--	0.31	3.45	6.26
		Off-Scar	36.89	1	40.88	0.6	0.87	0.93	--	0.51	6.96	4.31
Blend 2	10.15 (Mode 1)	On-Scar	24.42	2.98	46.79	0.82	11.24	2.06	--	--	--	11.6
		Off-Scar	34.09	1.94	42.55	0.18	1.29	1.3	--	0.98	6.77	4.07
Blend 2	10.15 (Mode 2)	On-Scar	26.29	2.85	45.67	1.08	10.07	2.56	--	0.2	0.82	9.66
		Off-Scar	40.22	1.39	39.65	0.22	0.77	1.07	0.15	0.72	6.66	2.5
Blend 3	1.22	On-Scar	36.86	2.04	41.19	0.46	4.85	3.71	0.24	0.34	2.28	5.76
		Off-Scar	46.79	1.67	38.82	0.15	0.4	0.93	--	0.49	4.37	1.91
Blend 3	10.15	On-Scar	23.12	2.54	48.55	0.71	10.11	2.36	0.23	0.14	0.17	11.91
		Off-Scar	39.26	3.91	39.51	0.2	0.4	1.5	0.72	0.51	4.51	4.95
Blend 4	1.22	On-Scar	36.36	2.84	44.09	0.45	0.94	0.46	--	0.73	6.96	0.16
		Off-Scar	43.36	3.97	39.13	--	--	0.39	--	1.12	5.95	--
Blend 5	1.22	On-Scar	57.04	1.38	32.11	0.96	0.99	--	--	0.31	3.56	--
		Off-Scar	43.1	3.07	41.13	--	0.18	--	0.14	0.45	5.9	--
Blend 6	1.22	On-Scar	41.23	2.68	36.87	0.71	2.3	2.4	0.14	0.78	3.69	5.51
		Off-Scar	43.64	3.28	36.66	0.38	0.11	0.25	--	0.53	7.15	0.79
Blend 6	7.49 (Mode 1)	On-Scar	49.26	4.87	24.04	1.74	11.4	6.39	0.15	0.5	0.58	0.47
		Off-Scar	56.46	5.17	30.77	0.24	0.41	0.56	0.13	0.87	2.62	0.14
Blend 6	7.49 (Mode 2)	On-Scar	40.8	5.16	40.2	0.52	7.49	4.03	--	--	0.45	0.86
		Off-Scar	49.82	4.6	33.8	0.38	0.45	0.12	--	0.75	4.61	0.81
Blend 6	10.15	On-Scar	40.21	4.44	37.33	0.99	8.41	3.96	--	0.41	0.92	2.4
		Off-Scar	49.08	5.37	34.6	0.15	0.31	1.01	--	0.66	4.06	0.64
Blend 7	1.22	On-Scar	45.84	3.47	36.72	0.67	4.2	2.47	--	0.78	1.59	2.69
		Off-Scar	49.71	2.24	35.48	0.52	0.43	0.32	--	0.83	4.77	0.91
Blend 7	7.49 (Mode 1)	On-Scar	37.22	2.37	40.31	0.29	7.39	2.88	--	0.41	1.22	6.64
		Off-Scar	55.08	2.46	32.84	0.17	0.4	0.39	0.14	0.59	3.56	0.81
Blend 7	7.49 (Mode 2)	On-Scar	27.41	1.5	43.52	0.59	8.34	2.98	--	0.39	1.36	12.53
		Off-Scar	36.24	1.38	41.85	0.24	0.16	0.15	0.15	0.36	8.83	1.79
Blend 7	10.15 (Mode 1)	On-Scar	34.85	1.68	41.21	0.49	7.46	2.62	--	0.39	1.39	8.51
		Off-Scar	49.19	2.84	34.44	0.21	0.35	0.64	--	0.55	5.29	1.16
Blend 7	10.15 (Mode 2)	On-Scar	30.29	1.58	43.75	0.56	7.94	2.62	0.11	0.4	2.15	8.48
		Off-Scar	50.28	2.69	35.23	0.35	0.44	0.95	--	0.5	3.99	1.56

SII. Chemical composition

On- and off-scar surface chemical compositions for various blends and loads are listed in the following tables.

Blend 1 (base oil)

		On-Scar Analysis			Off-Scar Analysis	
Load (kg)	Element	Energy (eV)	Peak Area (%)	Chemical State	Energy (eV)	Peak Area (%)
1.22	C	284.44	64%	Hydrocarbon	284.73	60%
		286.29	22%	Alcohol/ether	286.71	21%
		288.27	14%	Carboxyl	288.52	19%
	S	162.01	0%	Sulfide, mercaptan	163.75	24%
		168.86	100%	Sulfate, sulfone	169.01	76%
	Fe	710.28	100%	Iron oxides, hydroxides	710.18	100%
2.45	C	284.76	74%	Hydrocarbon	284.83	63%
Mode 1		286.54	19%	Alcohol/ether	286.49	16%
		288.64	7%	Carboxyl	288.51	21%
	S	162.39	30%	Sulfide, mercaptan	161.92	6%
		168.82	70%	Sulfate, sulfone	168.88	94%
	Fe	711.44	100%	Iron oxides, hydroxides	710.48	100%
2.45	C	284.74	71%	Hydrocarbon	284.85	57%
Mode 2		286.53	16%	Alcohol/ether	286.59	17%
		288.52	13%	Carboxyl	288.71	27%
	S	162.62	10%	Sulfide, mercaptan	162.01	0%
		168.9	90%	Sulfate, sulfone	168.67	100%
	Fe	710.97	100%	Iron oxides, hydroxides	710.73	100%

Blend 2 (base oil + 0.05 wt% ZDDP)

Load (kg)	Element	On-Scar Analysis Energy (eV)	On-Scar Analysis Peak Area (%)	Chemical State	Off-Scar Analysis Energy (eV)	Off-Scar Analysis Peak Area (%)
1.22	C	284.5	61%	Hydrocarbon	284.78	59%
		285.71	26%	Alcohol/ether	286.43	20%
		288.58	13%	Carboxyl	288.63	21%
	S	162.07	84%	Sulfide, mercaptan	162.4	39%
		169.58	16%	Sulfate, sulfone	169.04	61%
	Fe	710.66	100%	Iron oxides, hydroxides	710.49	100%
10.15 Mode 1	C	284.77	73%	Hydrocarbon	284.72	54%
		286.26	22%	Alcohol/ether	286.25	22%
		287.54	5%	Carboxyl	288.55	24%
	S	162.49	100%	Sulfide, mercaptan	162.17	50%
		168.86	0%	Sulfate, sulfone	168.91	50%
	Fe	710.48	0%	Iron oxides, hydroxides	710.5	100%
10.15 Mode 2	C	284.81	71%	Hydrocarbon	284.75	60%
		286.4	26%	Alcohol/ether	286.34	17%
		288.9	3%	Carboxyl	288.61	23%
	S	162.55	98%	Sulfide, mercaptan	162.45	45%
		169.24	2%	Sulfate, sulfone	168.79	55%
	Fe	711.74	100%	Iron oxides, hydroxides	710.41	100%

Blend 3 (base oil + 0.08 wt% ZDDP)

Load (kg)	Element	On-Scar Analysis Energy (eV)	On-Scar Analysis Peak Area (%)	Chemical State	Off-Scar Analysis Energy (eV)	Off-Scar Analysis Peak Area (%)
1.22	C	284.59	57%	Hydrocarbon	284.91	58%
		285.85	27%	Alcohol/ether	286.98	15%
		288.63	16%	Carboxyl	288.83	27%
	S	161.91	91%	Sulfide, mercaptan	161.94	49%
		169.81	9%	Sulfate, sulfone	168.71	51%
	Fe	710.69	100%	Iron oxides, hydroxides	710.1	100%
10.15	C	284.76	62%	Hydrocarbon	284.77	51%
		286.25	30%	Alcohol/ether	286.35	25%
		288.13	8%	Carboxyl	288.7	24%
	S	162.38	100%	Sulfide, mercaptan	162.28	67%
		167.16	0%	Sulfate, sulfone	168.74	33%
	Fe	710.99	100%	Iron oxides, hydroxides	710.79	100%

Blend 4 (base oil + 0.1 wt% dispersant A)

		On-Scar Analysis			Off-Scar Analysis	
Load (kg)	Element	Energy (eV)	Peak Area (%)	Chemical State	Energy (eV)	Peak Area (%)
1.22	C	284.81	64%	Hydrocarbon	284.64	56%
		286.87	19%	Alcohol/ether	286.41	16%
		288.55	17%	Carboxyl	288.2	27%
	S	162.54	15%	Sulfide, mercaptan	162.04	44%
		168.55	85%	Sulfate, sulfone	168.81	56%
	Fe	710.33	100%	Iron oxides, hydroxide[illegible]	709.68	100%

Blend 5 (base oil + 0.1 wt% dispersant B)

		On-Scar Analysis			Off-Scar Analysis	
Load (kg)	Element	Energy (eV)	Peak Area (%)	Chemical State	Energy (eV)	Peak Area (%)
1.22	C	284.77	54%	Hydrocarbon	284.51	51%
		286.27	33%	Alcohol/ether	286.19	29%
		288.53	13%	Carboxyl	288.3	20%
	S	162.01	0%	Sulfide, mercaptan	162.01	0%
		168.86	100%	Sulfate, sulfone	168.84	100%
	Fe	710.24	100%	Iron oxides, hydroxides	709.86	100%

Blend 6 (base oil + 0.1 wt% dispersant A)

		On-Scar Analysis			Off-Scar Analysis	
Load (kg)	Element	Energy (eV)	Peak Area (%)	Chemical State	Energy (eV)	Peak Area (%)
1.22	C	284.82	67%	Hydrocarbon	284.82	56%
		286.51	17%	Alcohol/ether	286.44	22%
		288.67	16%	Carboxyl	288.57	21%
	S	161.39	95%	Sulfide, mercaptan	162.01	0%
		168.67	5%	Sulfate, sulfone	168.67	100%
	Fe	710.44	100%	Iron oxides, hydroxides	710.28	100%
7.49	C	284.47	69%	Hydrocarbon	284.38	52%
Mode 1		286.07	27%	Alcohol/ether	286.02	31%
		287.9	3%	Carboxyl	288.02	17%
	S	161.42	97%	Sulfide, mercaptan	162.38	21%
		169.35	3%	Sulfate, sulfone	168.12	79%
	Fe	710.5	100%	Iron oxides, hydroxides	709.34	100%
7.49	C	284.28	51%	Hydrocarbon	284.79	59%
Mode 2		285.76	40%	Alcohol/ether	286.31	20%
		287.88	9%	Carboxyl	288.45	21%
	S	161.54	100%	Sulfide, mercaptan	162.02	92%
		168.86	0%	Sulfate, sulfone	168.83	8%
	Fe	710.5	100%	Iron oxides, hydroxides	710.31	100%
10.15	C	284.54	57%	Hydrocarbon	284.83	57%
		286.18	32%	Alcohol/ether	286.58	26%
		288.3	11%	Carboxyl	288.5	17%
	S	161.7	97%	Sulfide, mercaptan	162.23	63%
		166	3%	Sulfate, sulfone	168.4	37%
	Fe	710.5	100%	Iron oxides, hydroxides	710.06	100%

Blend 7 (base oil + 0.05 wt% ZDDP + 0.1 wt% dispersant B)

		On-Scar Analysis			Off-Scar Analysis	
Load (kg)	Element	Energy (eV)	Peak Area (%)	Chemical State	Energy (eV)	Peak Area (%)
1.22	C	284.63	66%	Hydrocarbon	284.81	57%
		286.24	24%	Alcohol/ether	286.43	24%
		288.28	10%	Carboxyl	288.51	19%
	S	161.67	93%	Sulfide, mercaptan	162.92	38%
		168.76	7%	Sulfate, sulfone	168.75	63%
	Fe	710.71	100%	Iron oxides, hydroxides	710.15	100%
7.49 Mode 1	C	284.33	56%	Hydrocarbon	284.69	56%
		285.8	36%	Alcohol/ether	286.35	28%
		287.84	8%	Carboxyl	288.49	16%
	S	161.47	99%	Sulfide, mercaptan	162.35	33%
		168.6	1%	Sulfate, sulfone	168.1	67%
	Fe	710.56	100%	Iron oxides, hydroxides	710.14	100%
7.49 Mode 2	C	284.79	77%	Hydrocarbon	284.8	62%
		286.45	19%	Alcohol/ether	286.55	14%
		288.12	4%	Carboxyl	288.56	23%
	S	162.17	93%	Sulfide, mercaptan	162.01	0%
		169.08	7%	Sulfate, sulfone	168.86	100%
	Fe	710.59	100%	Iron oxides, hydroxides	710.43	100%
10.15 Mode 1	C	284.68	63%	Hydrocarbon	284.79	64%
		286.22	32%	Alcohol/ether	286.54	16%
		288.28	5%	Carboxyl	288.63	21%
	S	162.27	91%	Sulfide, mercaptan	162.64	16%
		168.6	9%	Sulfate, sulfone	168.86	84%
	Fe	710.97	100%	Iron oxides, hydroxides	710.7	100%
10.15 Mode 2	C	284.84	69%	Hydrocarbon	284.76	53%
		286.41	27%	Alcohol/ether	286.38	28%
		288.11	4%	Carboxyl	288.55	20%
	S	162.02	95%	Sulfide, mercaptan	163.33	33%
		168.47	5%	Sulfate, sulfone	168.99	67%
	Fe	711.12	100%	Iron oxides, hydroxides	710.51	100%

SIII. Elemental depth profiles

Elemental depth profiles for various blends and loads are shown in this section. The table below shows the legend of detected elements in the tribofilms.

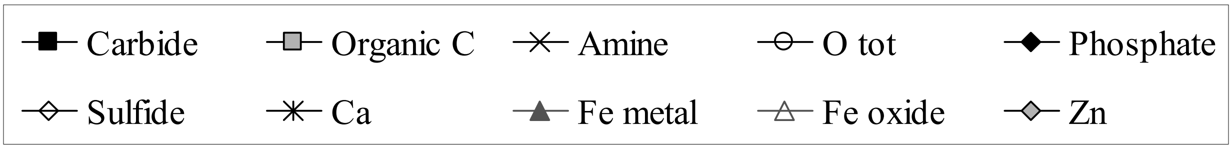

Blend 1

Load = 1.22 kg

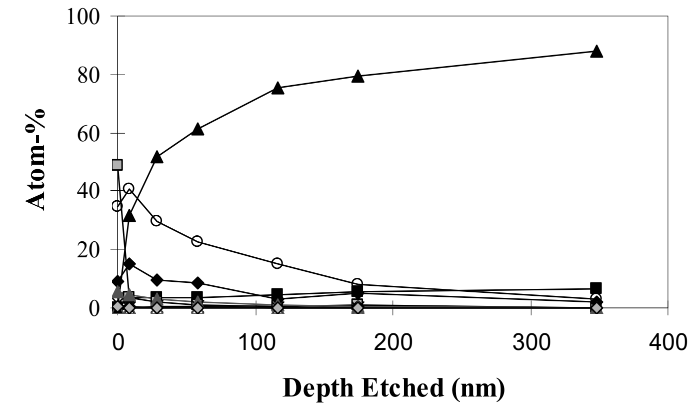

Load = 2.45 kg (mode M1)

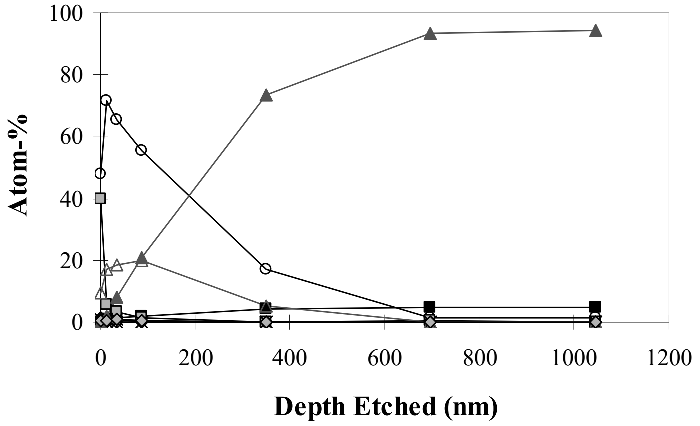

Load = 2.45 kg (mode M2)

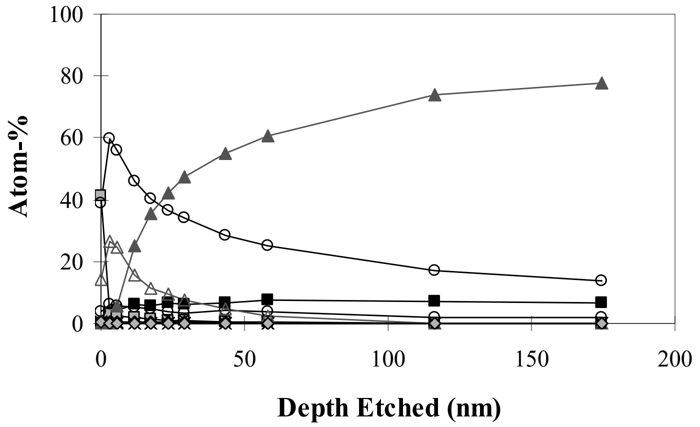

<u>Blend 2</u>

Load = 1.22 kg

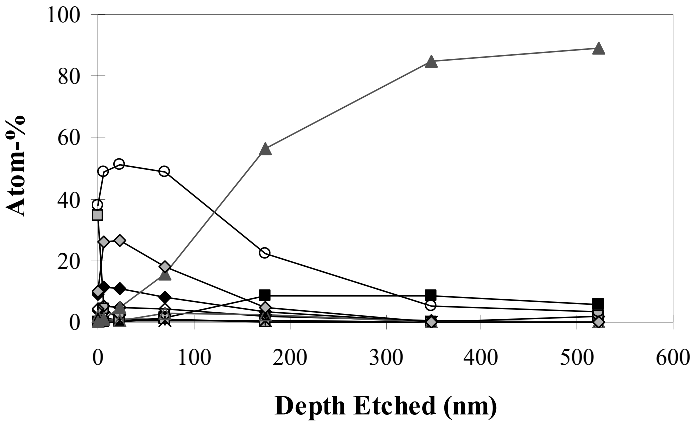

Load = 10.15 kg (mode M1)

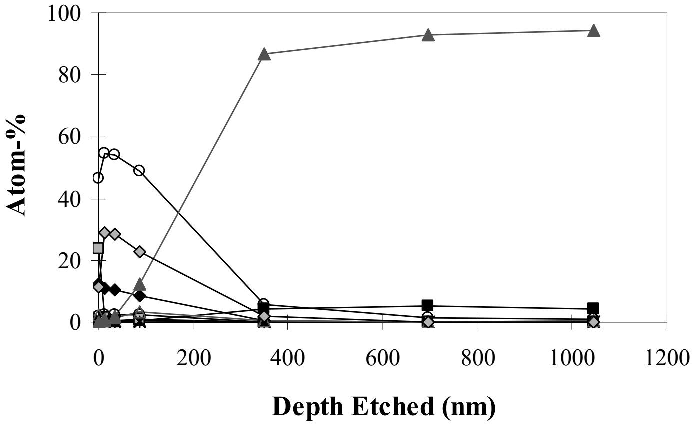

Load = 10.15 kg (mode M2)

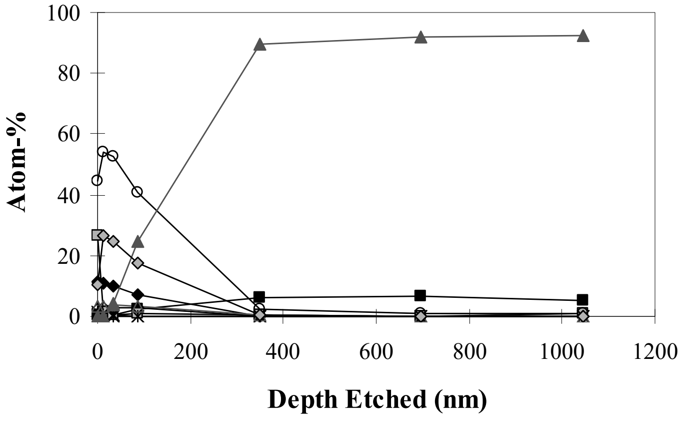

Blend 3

Load = 1.22 kg

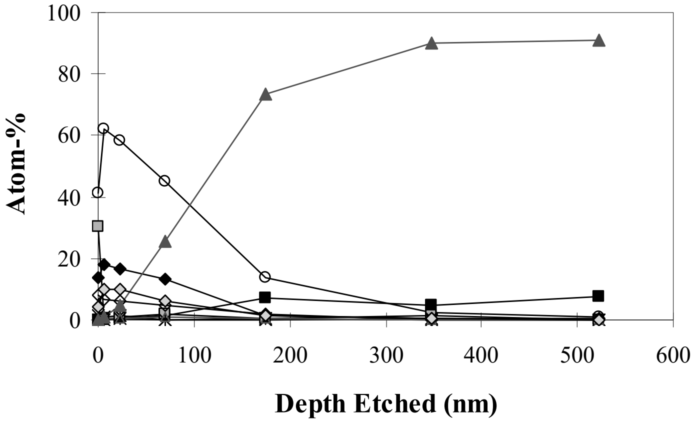

Load = 10.15 kg

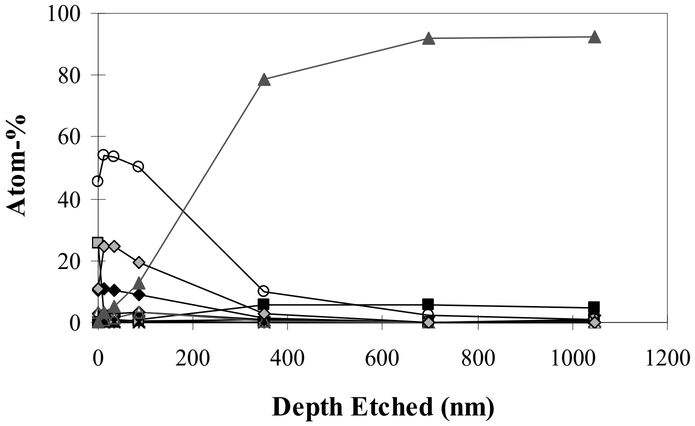

Blend 4

Load = 1.22 kg

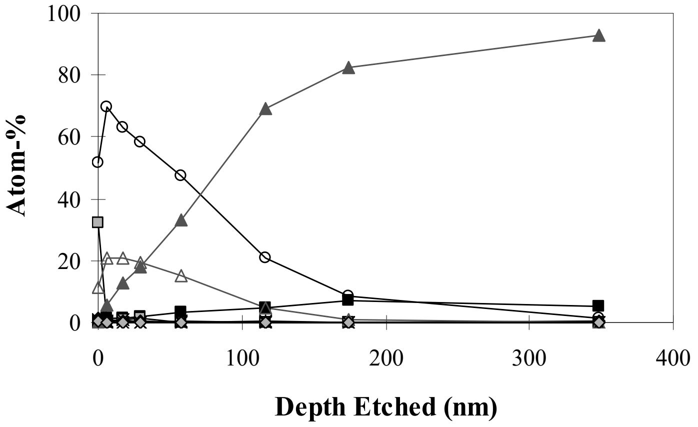

Blend 5

Load = 1.22 kg

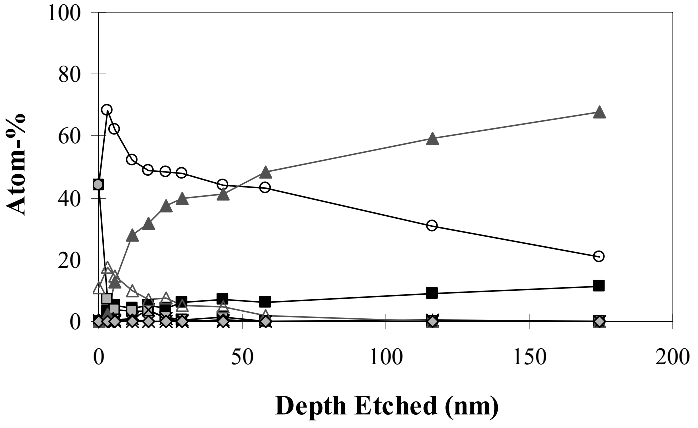

Blend 6

Load = 1.22 kg

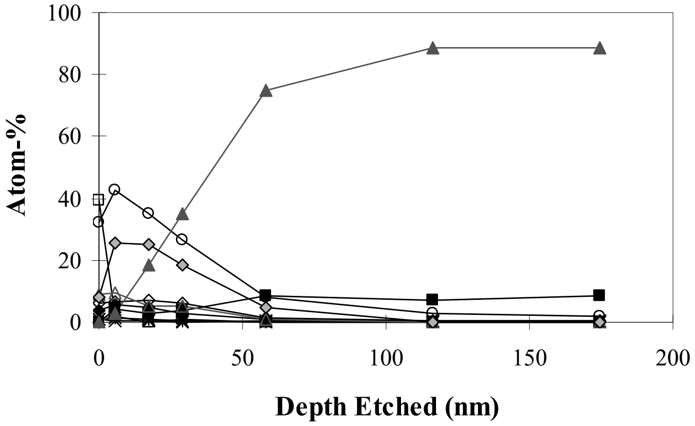

Load = 7.49 kg (mode M1)

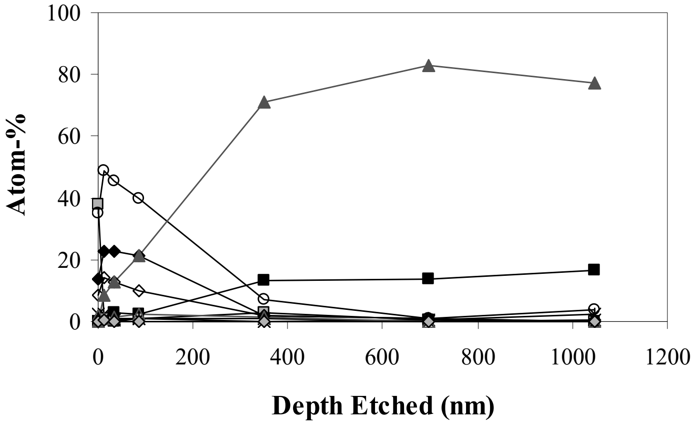

Load = 7.49 kg (mode M2)

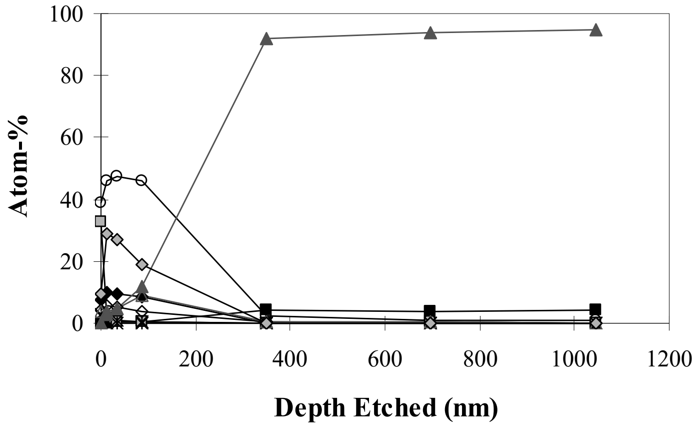

Load = 10.15 kg

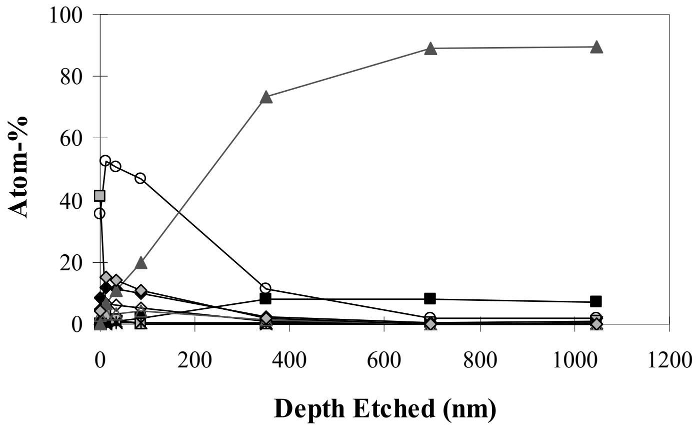

Blend 7

Load =1.22 kg

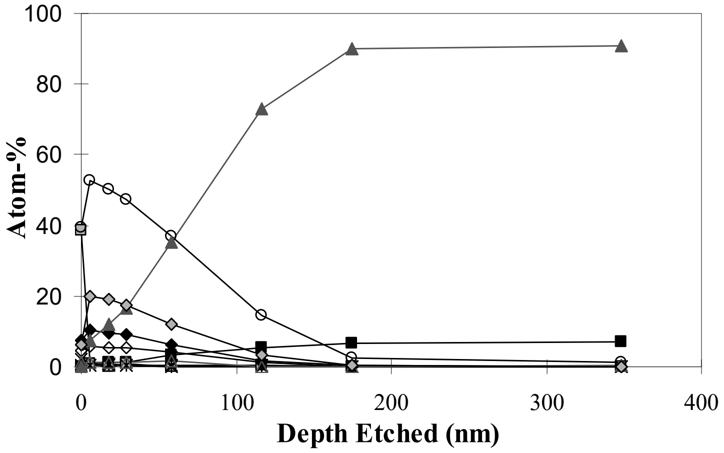

Load = 7.49 kg (mode M1)

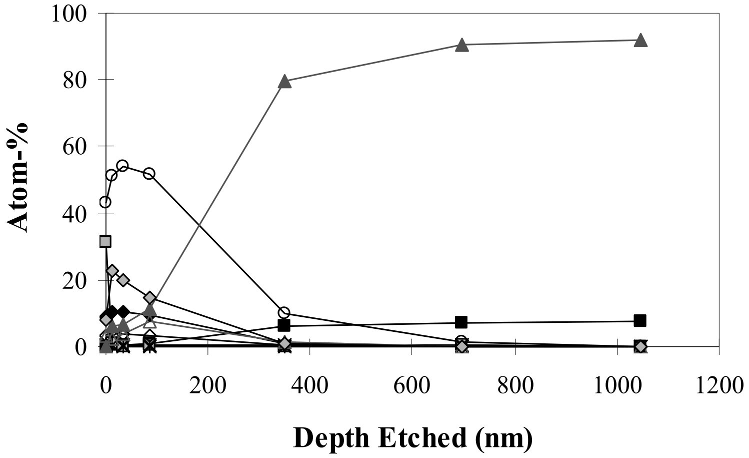

Load = 7.49 kg (mode M2)

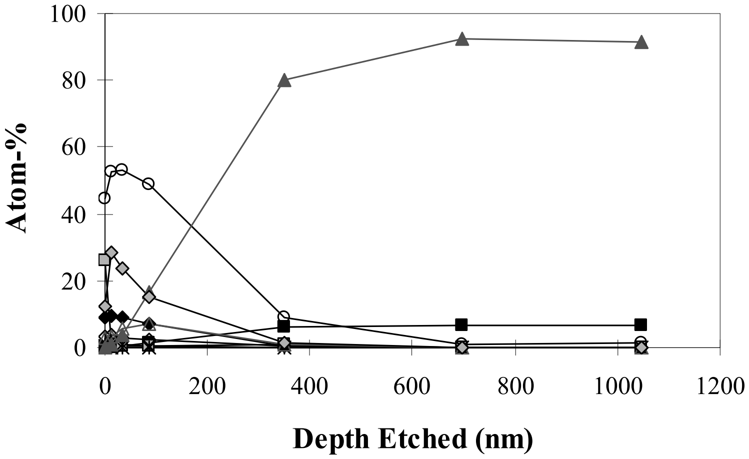

Load = 10.15 kg (mode M1)

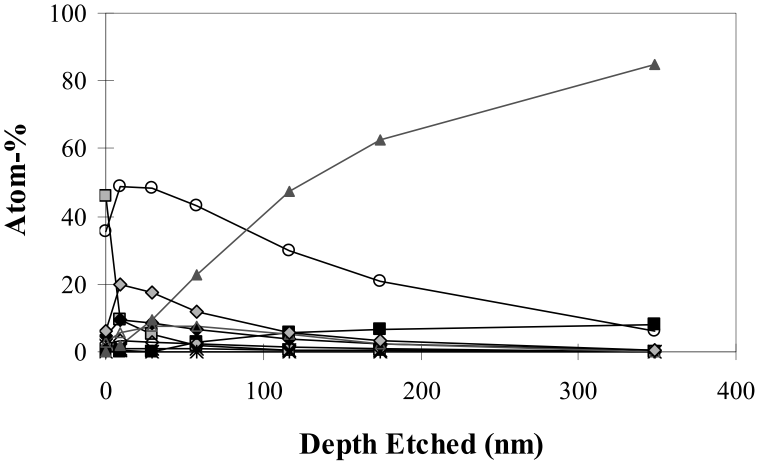

Load = 10.15 kg (mode M2)

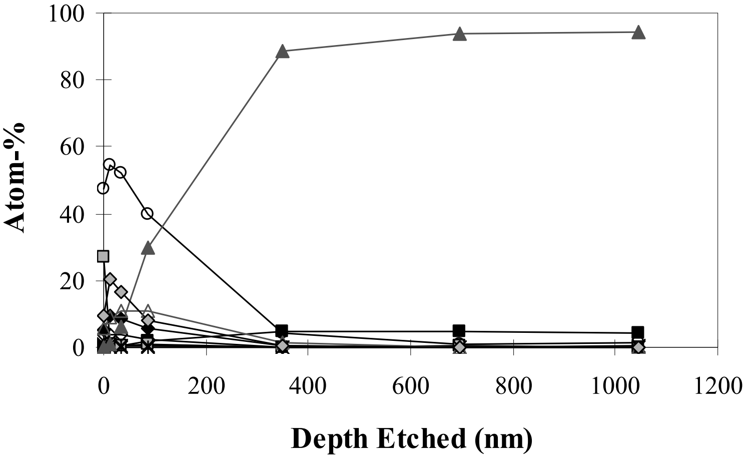
100
80
60
40
20
0
Atom-%
0
200
400
600
800
1000
1200
Depth Etched (nm)
